# Rather than by direct acquisition via lateral gene transfer, GHF5 cellulases were passed on from early Pratylenchidae to root-knot and cyst nematodes

**DOI:** 10.1186/1471-2148-12-221

**Published:** 2012-11-21

**Authors:** Katarzyna Rybarczyk-Mydłowska, Hazel Ruvimbo Maboreke, Hanny van Megen, Sven van den Elsen, Paul Mooyman, Geert Smant, Jaap Bakker, Johannes Helder

**Affiliations:** 1 Laboratory of Nematology, Department of Plant Sciences, Wageningen University, Droevendaalsesteeg 1, 6708 PB, Wageningen, The Netherlands; 2 Mathematisch-Naturwissenschaftliche Fakultät I, Institut für Biologie, Ökologie, Unter den Linden 6, Berlin, 10099, Germany

**Keywords:** Lateral gene transfer, Cellulase, Nematodes, Plant parasitism

## Abstract

**Background:**

Plant parasitic nematodes are unusual Metazoans as they are equipped with genes that allow for symbiont-independent degradation of plant cell walls. Among the cell wall-degrading enzymes, glycoside hydrolase family 5 (GHF5) cellulases are relatively well characterized, especially for high impact parasites such as root-knot and cyst nematodes*.* Interestingly, ancestors of extant nematodes most likely acquired these GHF5 cellulases from a prokaryote donor by one or multiple lateral gene transfer events. To obtain insight into the origin of GHF5 cellulases among evolutionary advanced members of the order Tylenchida, cellulase biodiversity data from less distal family members were collected and analyzed.

**Results:**

Single nematodes were used to obtain (partial) genomic sequences of cellulases from representatives of the genera *Meloidogyne, Pratylenchus, Hirschmanniella* and *Globodera.* Combined Bayesian analysis of ≈ 100 cellulase sequences revealed three types of catalytic domains (A, B, and C). Represented by 84 sequences, type B is numerically dominant, and the overall topology of the catalytic domain type shows remarkable resemblance with trees based on neutral (= pathogenicity-unrelated) small subunit ribosomal DNA sequences. Bayesian analysis further suggested a sister relationship between the lesion nematode *Pratylenchus thornei* and all type B cellulases from root-knot nematodes. Yet, the relationship between the three catalytic domain types remained unclear. Superposition of intron data onto the cellulase tree suggests that types B and C are related, and together distinct from type A that is characterized by two unique introns.

**Conclusions:**

All Tylenchida members investigated here harbored one or multiple GHF5 cellulases. Three types of catalytic domains are distinguished, and the presence of at least two types is relatively common among plant parasitic Tylenchida. Analysis of coding sequences of cellulases suggests that root-knot and cyst nematodes did not acquire this gene directly by lateral genes transfer. More likely, these genes were passed on by ancestors of a family nowadays known as the Pratylenchidae.

## Background

Any movement of genetic information, other than by vertical transmission from parents to their offspring via conventional reproduction, is defined as horizontal or lateral gene transfer (HGT or LGT). Although LGT occurs frequently among members of the Archaea and Bacteria, there are only a few probable cases of LGT between prokaryotes and multicellular eukaryotes that have resulted in new functional genes for the recipient. Likely cases of LGT in which the eukaryote is acting as a donor have been described for two mosquito species, *Aedes aegypti* and *Anopheles gambiae*[1]. The transfer of a gene related to malaria sporozoite invasion from mosquito to its endosymbiotic bacterium *Wolbachia pipientis* was demonstrated by Woolfit et al. (2008, [1]). This gene showed substantial divergence, and the level of expression suggested it to be functional in the new prokaryote host. Inter-domain gene transfers can also happen in the reverse way. The pea aphid *Acyrthisiphon pisum* probably acquired two genes from bacteria by LGT [2]. These laterally transferred genes are expressed in the bacteriocytes, and they contribute to the maintenance of *Buchnera aphidicola,* the aphid’s primary symbiont. Donors from multiple domains (bacteria, fungi and plants) are thought to be implicated in the acquisition of at least ten protein-coding sequences by the bdelloid rotifer *Adineta vaga*[3]. A subset of these genes were transcribed and correctly spliced. Interestingly, the authors hypothesized that LGT could be facilitated by mechanisms underlying the desiccation tolerance of this rotifer.

The lateral gene transfer of prokaryotic genes has presumably also played a key role in the evolution of plant parasitism in nematodes. Plant cells are protected by a cell wall, and penetration of this wall is a prerequisite to reach the cytosol. Potato and a soybean cyst nematode (*Globodera rostochiensis* and *Heterodera glycines*) were the first animals shown to harbor symbiont-independent cellulases [4]. These cellulases are classified as members of the glycoside hydrolase family 5 (GHF5). The nematode cellulases appeared to be most similar to bacterial cellulases. In an editorial comment Keen and Roberts [5] suggested that lateral gene transfer may drive the mobility of “pathogenicity islands” (including cellulases) from one organism to the other. Over the last decade, plant parasitic nematodes were shown to harbor a wide spectrum of cell wall-degrading proteins such as pectate lyases [6], polygalacturonase [7], xylanases [8] and expansins [9]. These genes are expressed during infective life stages, and contribute to nematode’s ability to exploit plants as a food source.

Bacterivory is generally accepted as the ancestral feeding type of nematodes. A longstanding hypthesis suggests that bacterivores gave rise to fungivorous nematodes, and facultative and obligatory plant parasites arose from fungal feeding ancestors [10]. It is conceivable that the evolution of plant parasitism in nematodes was driven by the lateral transfer of genes via ingestion of the donor (soil bacteria) by the recipient (bacterivorous nematodes) [11]. Mechanisms underlying desiccation tolerance could have facilitated the uptake of prokaryotic DNA [12]. A number of nematode species including *Aphelenchus avenae*[13], *Ditylenchus dipsaci*[14], and *Panagrolaimus superbus*[15] can develop into highly drought resistant Dauer larva.

Among nematode genes that could have been acquired via one or multiple HGT events, GHF5 cellulases are best characterized. Recent genome sequencing projects resulted in the identification of large cellulase families in the root-knot nematodes *Meloidogyne incognita*[16,17] and *Meloidogyne hapla*[18]. These are highly derived (distal) species within the family Meloidogynidae, and to identify possible origin(s) of these genes, cellulase sequence information is required from less derived representatives of this family. Recent morphological and molecular studies based on female gonoduct architecture [19] and small subunit ribosomal DNA sequences [19,20] suggest that root-knot nematodes originate from - and constitute a subclade within - the genus *Pratylenchus*. By sequencing cellulase genes from *Pratylenchus spp.* (lesion nematodes) and basal root-knot nematode species - the ones that do not belong to one of the subclades I, II and III as defined in 2002 by Tandingan De Ley *et al.*[21] -, we intended to generate clues to establish the evolutionary relationship between members of the Pratylenchidae genera *Pratylenchus* and *Hirschmanniella,* and basal root-knot nematode species such as *Meloidogyne ichinohei, M. mali* and *M ulmi*[20].

Several models have been proposed about HGT event(s) underlying the acquisition of cellulases by plant parasitic and fungivorous nematodes. So far it is unclear whether the distribution of cellulase-encoding genes among Tylenchida is the result of a single HGT event, followed by early single duplication event as suggested by Kyndt et al. [22], or the outcome of multiple HGT events. Comparison of the topologies of phylogenetic trees based on SSU rDNA data (*e.g.*[20,23]) *versus* GHF5 cellulase-based tree might tell us whether the evolution within the Pratylenchidae/Meloidogynidae branch includes one or multiple distinct cellulase lineages. Analysis of 103 paralogs and orthologs of cellulase-encoding gene(s) (fragments) from plant parasitic Tylenchida revealed a major clade with a topology similar to the one revealed by SSU rDNA, a neutral gene. Moreover, a relatively small, divergent subset of cellulases was found that is probably the result of early substrate specificity-driven diversification. Within the catalytic domain types A and B (too few type C sequences are available to make a statement), the overall topology resembles the topologies revealed by neutral ribosomal DNA sequences, and it is hypothesized that root-knot, cyst and lesion nematodes received their cellulases from more ancient Pratylenchidae or even more basal members of Clade 12 [24], rather than by direct lateral gene transfer.

## Methods

### Taxon sampling and microscopic identification

*Pratylenchus* and *Hirschmanniella* species were collected from various habitats throughout The Netherlands and extracted from the soil using standard techniques. Individual nematodes were identified using a light microscope (Zeiss Axioscope) equipped with differential interference contrast optics. *Globodera pallida* specimens originated from a Dutch population named Pa3 – Rookmaker. *Meloidogyne* species were kindly provided by Dr. Gerrit Karssen from the Plant Protection Service of The Netherlands: *M. ichinochei* (propagated on *Iris levigata;* culture C2312; Japan), *M. artiellia* (sampled from a field with *Triticum aestivum*; culture E8067; Syria), *M. ardenensis* (propagated on *Liguster sp.*; Wageningen) and *M. ulmi* (isolated from *Ulmus* sp.; Wageningen).

### Nematode lysis

Single nematodes were transferred to a 0.2 mL polymerase chain reaction (PCR) tube containing 25 μL sterile water. An equal volume of lysis buffer containing 0.2 M NaCl, 0.2 M Tris–HCl (pH 8.0), 1% (v/v) β-mercaptoethanol and 800 μg/mL proteinase K was added. Lysis took place in a Thermal cycler (MyiQ, Bio-Rad) at 65°C for 2 h followed by 5 min incubation at 100°C. The lysate (crude DNA extract) was used immediately or stored at −20°C.

### Amplification of cellulase-coding genes from genomic DNA

Based on publicly available cellulase sequences (cDNA and genomic sequences, see Additional file 1: Table S1) from lesion, root-knot, and cyst nematodes, six conserved peptide motives were identified within the catalytic domain, namely PPYGQLS (CD1), LKCNWN (CD2), YVIVDW (ENG1), WCQDV (CD4), FVTEYG (ENG2) and ISYLNWAISD (CD6) (for positioning see Table 1). These regions were used as a starting point for the design of *eng*-specific primers (Table 1). The primary aim was to amplify the longest possible fragment, preferably from CD1 to CD6 (230 amino acids, ≈700bp of the coding sequence); however, on some occasions, only shorter cellulase fragments could be amplified (Table 2).

**Table 1:** Overview of PCR primers used for cellulase amplication from individual nematodes

**A**			
**CD1**	**CD2**		
TATP**PPYGQLS**VSGTKLVDSSGQPVQLIGNSLFWHQFQAQYWNAETVKA**LKCNWN**ANVVRAAVGVDLERGYMSDP			
**ENG1**			
TTAYNQAVAVIEAAISQGL**YVIVDW**HSHESHVDKAIEFFTKIAKAYGSYPHVLYETFNEPLQGVSWTDILVPYHKKVIAAI			
**CD4**	**ENG2**		
RALDSKNVIILGTPT**WCQDV**DIASQNPIKEYKNLMYTFHFYAATHFVNGLGAKLQTAINNGLPI**FVTEYG**TCSADGNGNI			
**CD6**			
DTNSISSWWSLMDNLK**ISYLNWAISD**KSETCSALKPGTPAANVGVSSSWTTSGNMVADHDKKKSTGVSCS			
B Primer sequence 5’→3’ *		Primer sequence 5’→3’ *	
**Region PPYGQLS (CD1):**		**Region WCQDV (CD4):**	
CD1aF	cc**I**cc**I**ta**c**gg**I**caatt**g**tc	CD4aR	tccac**R**tc**c**tgg**g**acca
CD1bF	cc**I**cc**I**ta**t**gg**I**caatt**g**tc	CD4cR	tccac**A**tc**t**tgg**c**acca
CD1cF	cc**I**cc**I**ta**t**gg**I**caatt**a**tc		
CD1PraFa	cc**g**cc**g**ta**t**gg**g**caa	**Region FVTEYG (ENG2):**	
CD1PraFb	cc**t**cc**c**ta**t**gg**c**caa	ENG2 see *e.g.*[22]	gtIccRtaYTcIgtIacRaa
CD1PraFc	c**g**cc**g**ta**t**gg**g**caa		
CD1MelF	c**t**cc**a**ta**t**gg**g**caatt**a**tctgt	**Region ISYLNWAISD (CD6):**	
**Region LKCNWN (CD2):**		CD6PraFb	tctcctacatcaactgggc
CD2aF	ct**c**aaatgc**aa**ttggaa**c**Kc	CD6aR	gccca**g**ttg**gcg**ta**I**ga**g**a
CD2bF	ct**c**aaatgc**aa**ttggaa**t**Kc	CD6bR	gccca**I**ttg**gcR**ta**I**ga**a**a
CD2cF	ct**t**aaatgc**aI**ttggaa**t**Kc	CD6cR	gccca**I**ttg**aIg**ta**M**ga**a**a
CD2dF	ct**t**aaatgc**tI**ttggaa**t**Kc	CD6dR	gccca**g**ttg**aYg**ta**I**ga**g**a
**Region YVIVDW (ENG1):**		CDGp8R	gccca**g**ttg**agg**ta**c**ga**a**
ENG1 see *e.g.*[22]	taYgtIatcgtIgaYtggca	CD6PraRa	ccca**g**ttg**gcg**ta**g**ga
ENG1R	tgccaRtcIacgatIacRta	CD6MelR	tgtttgagatagccca**g**ttg

* I=inosine; K= g or t, M= a or c, R= a or g, Y= c or t. In bold: discriminative nucleotide position.

Conserved amino acid motives in GHF5 cellulases from plant parasitic nematodes residing in nematode Clade 12 (Holterman *et al.* 2006 [24]). Primer design was based on these motives, and all primers used in this study are listed bellow. **A.** The backbone sequence given below is derived from the predicted amino acid sequence of the potato cyst nematode (*Globodera rostochiensis*) cellulase *Gr-eng*-1 (GenBank AF004523), amino acid positions 18 – 324 (mainly catalytic domain). Underlined: part of signal peptide for secretion. **B.** Primer names and primer sequences.

**Table 2:** Overview of GHF5 cellulase sequences generated in this study from plant parasitic nematodes belonging to the superfamily Hoplolaimoidea

**Species name**	Individual*	**Forward primer (1)**	**Reverse primer(1)**	**Fragment length (bp)**	**Fragment length after removal putative introns (bp)**	**Gene name (2)**	**GenBank Acc. No.**
*Meloidogyne ardenensis*	1	CD1cF	CD6cR	1228	**727**	***Mard-eng-1***	JN052024
*Meloidogyne artiellia*	1	CD1MelF	CD6MelR	936	**731**	***Mart-eng-1***	JN052025
*Meloidogyne ichinochei*	1	CD2aF	CD4aR	673	343	*Mic-eng-1*	JN052026
*M. ichinochei*	2	CD2aF	CD4aR	673	343	*Mic-eng-2*	JN052027
*Hirschmanniella gracilis*	1	CD1aF	CD6bR	950	**728**	***Hgr-eng-1***	JN052061
*H. gracilis*	1	CD1aF	CD6bR	866	**728**	***Hgr-eng-2***	JN052062
*H. gracilis*	1	CD1aF	CD6bR	950	**728**	***Hgr-eng-3***	JN052063
*Hirschmanniella loofi*	1	CD1bF	ENG1R	282	255	*Hl-eng-1*	JN052057
*H. loofi*	1	CD1bF	CD4aR	560	487	*Hl-eng-2*	JN052058
*H. loofi*	1	ENG1	CD6aR	983	461	*Hl-eng-3*	JN052059
*H. loofi*	1	ENG1	CD6aR	554	431	*Hl-eng-4*	JN052060
*Pratylenchus crenatus*	1	CD1PraFa	CD6aR	820	**721**	***Pcr-eng-1***	JN052031
*P. crenatus*	2	CD1PraFb	CD6PraRa	819	**723**	***Pcr-eng-2***	JN052030
*P. crenatus*	3	ENG1F	CD6aR	543	449	*Pcr-eng-3*	JN052029
*Pratylenchus neglectus*	1	ENG1	CD6aR	508	452	*Pn-eng-1*	JN052032
*P. neglectus*	2	CD1PraFc	CD6PraRa	791	**735**	***Pn-eng-2***	JN052033
*P. neglectus*	2	CD1PraFc	CD6PraRa	789	**733**	***Pn-eng-3***	JN052034
*Pratylenchus penetrans*	1	CD2cF	CD6aR	1514	588	*Pp-eng-3*	JN052035
*P. penetrans*	1	CD2cF	CD6aR	695	587	*Pp-eng-4*	JN052036
*P. penetrans*	1	CD1bF	CD4aR	739	484	*Pp-eng-5*	JN052037
*P. penetrans*	2	CD1PraFb	CD6PraRb	841	**733**	***Pp-eng-6***	JN052038
*Pratylenchus convalariae* (3)	1	CD1PraFb	CD6aR	874	**739**	***Pcon-eng-1***	JN052028
*Pratylenchus pratensis*	1	CD1bF	ENG1R	256	256	*Ppr-eng-1*	JN052040
*P. pratensis*	1	CD2bF	CD6dR	1124	592	*Ppr-eng-2*	JN052039
*P. pratensis*	1	CD2dF	CD4aR	407	349	*Ppr-eng-3*	JN052041
*P. pratensis*	1	CD1aF	CD4aR	489	489	*Ppr-eng-4*	JN052043
*P. pratensis*	1	ENG1	CD6aR	507	452	*Ppr-eng-5*	JN052042
*P. pratensis*	2	CD1PraFb	CD6PraRa	801	**750**	***Ppr-eng-6***	JN052044
*Pratylenchus thornei*	1	CD2bF	CD6dR	678	587	*Pt-eng-1*	JN052045
*P. thornei*	2	CD1PraFc	CD6PraRb	824	**733**	***Pt-eng-2***	JN052046
*Pratylenchus vulnus*	1	CD2cF	CD6aR	639	587	*Pv-eng-1*	JN052047
*P. vulnus*	1	CD2bF	CD6dR	1001	614	*Pv-eng-2*	JN052048
*P. vulnus*	1	CD2bF	CD6dR	639	587	*Pv-eng-3*	JN052049
*P. vulnus*	1	CD2aF	CD4aR	349	349	*Pv-eng-4*	JN052050
*P. vulnus*	1	ENG1	CD6aR	958	449	*Pv-eng-5*	JN052051
*P. vulnus*	1	ENG1	CD6aR	604	312	*Pv-eng-6*	JN052052
*P. vulnus*	1	ENG1	CD6aR	505	452	*Pv-eng-7*	JN052053
*P. vulnus*	2	CD1aF	CD6bR	1031	**728**	***Pv-eng-8***	JN052054
*P. vulnus*	3	CD1PraFb	CD6aR	790	**733**	***Pv-eng-9***	JN052055
*P. vulnus*	3	CD1PraFb	CD6PraRa	792	**735**	***Pv-eng-10***	JN052056
*Globodera pallida*	1	CD1aF	CD4cR	1144	481	*Gp-eng-1*	JN052064
*G. pallida*	1	CD2aF	CD6cR	852	589	*Gp-eng-2*	JN052065
*G. pallida*	1	CD2aF	CD6cR	853	590	*Gp-eng-3*	JN052066
*G. pallida*	2	CDGp2F	CD6cR	1561	**709**	***Gp-eng-4***	JN052067
*G. pallida*	2	CDGp2F	CDGp8R	706	**706**	***Gp-eng-5***	JN052068

Overview of GHF5 cellulase sequences generated in this study from plant parasitic nematodes belonging to the superfamily Hoplolaimoidea. Single nematodes were used for the amplification of putative cellulase fragments. Occasionally, multiple fragments were amplified from an individual, such as in case of *Pratylenchus neglectus* 2 (*Pn-eng-2 and −3*). (1) Primers sequences are given in Table 1. (2) gene names in bold indicate fragments spanning almost the full catalytic domain (CD1 - CD6). (3) Small subunit ribosomal DNA data suggest that *Pratylenchus convalariae* is identical to *P. penetrans*[20].

Due to differences in codon usage within the six conserved amino acid motives mentioned above, numerous primer variants had to be designed and subsequently examined in various combinations. For a quick, first selection of the most effective primer combinations, quantitative PCR was used. For this, 3 μL of template (single nematode lysate) was mixed with relevant primers (end concentrations for both primers 200 nM), and 12.5 μL iQ Absolute Sybr Green Fluorescein Cat. CM-225 (Westburg). The total reaction volume was 25 μL. Thermal cycling took place in the MyiQ thermal cycler (Bio-Rad) under the following conditions: 95°C for 15 min; followed by 60 cycles at 95°C for 30 s, 50°C for 1 min and 72°C for 2 min. In case a possibly applicable amplification signal was produced (criteria: C_t_ value < 50 cycles, and a melting temperature > 80°C), the amplicon was analyzed on a 1% agarose-gel stained with GelStar (Westburg; 2μl/100ml). For those primer combinations that gave rise to amplification of the expected size products, the annealing temperature was optimized using conventional PCR. These reactions were performed in a final volume of 25 μl and contained 3 μl of a diluted crude DNA extract, 0.1 μM of each PCR primer, and a Ready-To-Go PCR bead (Amersham, Little Chalfont, Buckinghamshire, UK). The following PCR profile was used: 95°C for 5 min followed by 60 x (94°C, 30 sec; specific annealing temperature, 1 min; 72°C, 2 min) and 72°C, 10 min.

### Cloning, sequencing and sequence alignment

Gel-purified amplification products (Marligen Bioscience, Ijamsville, MD) were cloned into a TOPO TA cloning vector (Invitrogen, Carlsbad, CA) and sequenced using standard procedures. Newly generated sequences were deposited at GenBank under accession numbers listed in Table 2.

Intron positions in the genomic sequences were identified on the basis of information about exon-intron structure of publicly available sequences (all the ones in the Additional file 1: Table S1 harboring at least one intron). The newly obtained nucleotide sequences as well as those derived from GenBank were translated into amino acids and aligned using the ClustalW algorithm as implemented in BioEdit 5.0.9 [25]. The protein alignment was improved manually and translated back into nucleotides. The final nucleotide alignment consisted of 103 sequences, of which 45 were generated in this study. More than half of these sequences (66 out of 103) span almost the full catalytic domain (from CD1 to CD6).

### RNA extraction and cDNA cellulase amplification

In order to support the chosen intron extraction approach, cDNA cellulase sequence for *G. pallida* was synthesized. For this purpose 100 individuals of *G. pallida* were collected into a 0.2 mL PCR tube containing 25 μL of sterile water and lysed as specified above. The lysate was used immediately for the RNA extraction according to RNeasy Micro kit protocol (Qiagen). Total RNA end concentration was approximately 7ng of RNA/μL of water. 3 μL of the five times diluted RNA was mixed with CDGp2F and CDGp8R primers (Table 1; end concentrations for both primers 200 nM), 20 units of RNAse inhibitor (Invitrogen) and the components of the SuperScript III One-Step RT-PCR with Platinium *Taq* kit (12 μL of 2X reaction Mix and 2 μL of the SuperScriptTM III RT/ Platinium *Taq* Mix). This reaction of 25 μL in total, was used for the specific cDNA fragment amplification under the following conditions: 60°C for 30 min, 94°C for 2 min; followed by 60 cycles at 94°C for 15 s, 60°C for 30 s and 68°C for 1 min and finished with one cycle of 68°C for 5 min. As a result of this experiment the Gp-eng-5 sequence was acquired.

### Phylogenetic analysis

The Bayesian phylogeny was constructed with the program MrBayes 3.1.2 using a site-specific model. Data were partitioned by codon position and gamma distribution of rate variation with a proportion of invariable sites was used. Four independent runs were set with 4 Markov chains per run. The program was run for 5 million generations. Stabilization of the likelihood and parameters were checked with the program Tracer v1.4 [26] and the burnin was defined as 120,000 generations. For the construction of the maximum likelihood of the cellulase tree the RAxML-HPC BlackBox program [27] available at the CIPRES Science Gateway V. 3.1 was used. The program FindModel (http://hcv.lanl.gov/content/sequence/findmodel/findmodel.html from the HCV sequence database) was used to determine the best phylogenetic model, and the following parameters were applied: estimated proportion of invariable sites (GTRGAMMA + I). To find best tree using maximum likelihood search, bootstrapping halted automatically and printed branch lengths. Cellulases from *Aphelenchus avenae,* a predominantly fungivorous species, were used as outgroup as this species does not belong to the Tylenchida (all other species used in this study belong to this order), and - on the basis of SSU rDNA data - resides at the very base of Cade 12.

## Results and discussion

### Identification of new cellulase genes in nematodes

Genomic DNA from individuals from seven *Pratylenchus* (Pratylenchidae), two *Hirschmanniella* (Pratylenchidae), three basal *Meloidogyne* (Meloidogynidae) and one *Globodera* (Heteroderidae) species were tested for the presence of GHF5 cellulases. In all of the nematodes we identified at least one GHF5 cellulase gene (Table 2). Recently, it was shown that GHF5 cellulases are not the only type of cellulases present among members of the Tylenchida (Bauters *et al.*, pers. comm.). Except for the very basal *Meloidogyne* species *M. ichinochei* and *Hirschmanniella loofi,* we obtained at least one complete CD1-CD6 (Table 2) sequence for every species under investigation.

**Figure 1:**
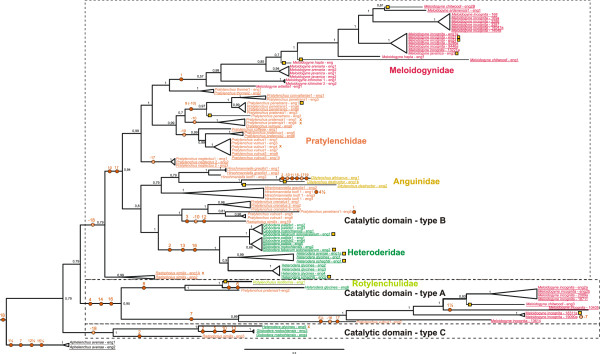
**Bayesian tree of GHF5 catalytic domains from members of the nematode order Tylenchida.** Genomic and coding sequences (indicated by a yellow box at the base of the relevant branch) from (partial) cellulase catalytic domains were analyzed. Sequences covering the catalytic domain from CD1 to CD6 (as defined in Table 1) are underlined (non underlined sequences are slightly shorter). Identical colors are used for members of the same nematode family. The tree is rooted with genomic cellulase sequences from the fungivorous nematode *Aphelenchus avenae* (infraorder Tylenchomorpha)*.* Posterior probabilities are given next to each node. Orange circles with or without a bright cross are used to indicate the presence or absence of an intron. An orange cross behind a sequence is used to indicate that the generated piece of a sequence was intronless. Intron numbering is essentially according to Kyndt et al. (2008) [22]. Branch length is calculated in MrBayes, and the scale bar below represents branch length (as number of DNA substitutions/site).

Genome sequencing of the distal tropical *Meloidogyne* species *M. incognita* not only revealed the presence of multiple (21) genes encoding putative cellulases, but also showed distinct clusters of GHF5 sequences within a single species [15]. Here we demonstrate that this is not unique for distal *Meloidogyne* species. *Pratylenchus pratensis* harbors multiple cellulase genes, whereas both type A or type B catalytic domains (as coined by [22]; Additional file 2: Table S2) are represented. Similar results were obtained for *Pratylenchus vulnus* (Figure 1)*.* The inventory of all currently available catalytic domain sequences points at a numerical dominance of the type B over type A and C catalytic domains. For the soybean cyst nematode *Heterodera glycines*, the substrate specificities of cellulases with type A, B and C catalytic domains were tested [28]. Hg-ENG5 (Type C) and Hg-ENG6 (Type A) differed greatly from the most abundant type B cellulases: their depolymerizing activity on carboxymethylcellulose was strongly reduced (respectively ≈40% and ≈20% of the activities of HG-ENG-1 and −4 (both belonging to type B cellulases)), whereas both degrade xylan and crystalline cellulose (Hg-ENG5 showing two fold higher activity than Hg-ENG6). By contrast, the latter two substrates were not significantly degraded by Hg-ENG-1 and −4 [28]. As such differences have been reported for other GHF5 cellulases as well (*e.g.*[29]), we hypothesize that the different types of catalytic domains could point at differences in substrate specificities.

### Phylogenetic analyses

Bayesian inference-based phylogenetic analysis of 103 coding sequences (of which 45 were generated in this study) for - at least - a major part of the catalytic domain of GHF5 cellulases resulted in the distinction of three major types of catalytic domains (A, B, and C, see Figure 1). This systematics elaborates on the evolutionary model proposed by Kyndt *et al.* (2008) [22]; catalytic domain C was originally presented as a well-supported group of cellulases nested within the type B clade [22].

Among cellulases with a type B catalytic domain (comprising 84 out of the 103 sequences), we observed a topology that shows a remarkable overall similarity with the one presented by Holterman *et al.* (2009) [20] on the basis of a neutral gene, *viz.* SSU rDNA. Also on the basis of type B catalytic domain sequences *M. artiellia* and *M. ichinochei* appear at the base of the family Meloidogynidae, and just as observed on the basis of SSU rDNA sequences the Meloidogynidae appear as an elaborate subclade nested within the genus *Pratylenchus.* Unlike SSU rDNA sequences that gave no clear answer about the nature of the link between the Pratylenchidae and the Meloidogynidae, a robust sister relationship was observed between a specific *Pratylenchus* species, *P. thornei*, and all representatives of the genus *Meloidogyne.*

Ribosomal DNA-based phylogenetic analysis revealed a sister relationship between lesion and root-knot nematodes on the one hand, and cyst nematodes, Hoplolaimidae and Rotylenchulidae on the other (*e.g.*[20]). Remarkably, all catalytic domains from the lesion nematode *P. crenatus,* and subsets from the *P. vulnus* and *P. penetrans* cellulases reside in a sister position with regard to all type B cyst nematode cellulases. Al-though the number of sequences included is considerably smaller, we observed a similar pattern for the type A catalytic domain. Firstly, a sister relationship was established here between all type A catalytic domain sequences from root-knot nematodes and a cellulase from *P. vulnus*, whereas a similar relationship was observed for a soybean cyst and a reniform nematode cellulase on the one hand, and *P. pratensis* on the other (Figure 1). Hence, at least some lesion nematode species are equipped with both root-knot and cyst nematode-like cellulases.

The positioning of *Ditylenchus spp.* is based only on one genomic and two CDSs sequences, and should be considered as a consequence of the virtual absence of cellulases data from other, more basal Tylenchida. The current positioning is not well supported, and will not be discussed.

### Exon-intron structure

The number of introns in the catalytic domains of the nematode cellulases varied between zero and seven. For the intron identifiers, we followed the nomenclature proposed by Kyndt et al. (2008) [22]. As a consequence of the increase in the number and the diversity of catalytic domains additional predicted intron positions were found. To label new intron positions without uprooting the existing systematics, we used identifiers such as 1½ and 5½ for introns positioned between introns 1 and 2, introns 5 and 6, *etc.* (see Additional file 2: Table S2)*.*

Most of the *in silico* predicted introns in the newly generated cellulase sequences were located at positions equivalent to the positions reported before for plant parasitic cyst and root-knot nematodes ( [30], [17], [28] and [22]) and the outgroup *Aphelenchus avenae*[31]. Identifiers 1½, 12½, 15½ were added as - up to now - unique introns in cellulase gDNAs from *A. avenae*, whereas 4½ and 5½ were used to indicate new intron positions in *Hl-eng1* and *Pv-eng2*. Among the newly generated cellulase sequences the largest intron was found in *Gp-eng1*; intron 2 with a length of 563 bp. Numerous occasions of intron gain and loss were observed in all three main types of cellulase catalytic domains tree (A, B and C). Particular introns appear to be characteristic for catalytic domain types: Type A typically contains introns 4 and 14, whereas all type B and C catalytic domains investigated so far have lost intron 18. Type C representatives share the presence of intron 2. However, this feature is not unique as it is typical for the type B cellulases from cyst nematodes as well. Among root-knot nematode type B cellulase the presence of intron 1 appears as a common characteristic. The two other cellulase catalytic domains with an intron at position 1 were found in a type B catalytic domain Pp-eng-5 from *Pratylenchus penetrans* and interestingly, in a type A catalytic domain Hg-eng-6 from soybean cyst nematodes.

### Intron phase distribution

The intron phase distribution in the catalytic domain of nematode GHF5 cellulases was biased towards phase 0; 16 out of the 24 introns (66%) were inserted in between two codons. Respectively, two and four phase 1 (after the first base of a codon) and phase 2 (after the second base of a codon) introns were identified, whereas intron positions 7 and 17 occurred in two phases (0 and 1). To some extend a bias towards the phase 0 introns was to be expected as the overall frequencies of intron phases 0, 1 and 2 in *Caenorhabditis elegans* are roughly 50%, 25% and 25% [32]. This phase bias seems to be stronger in the case of cellulase catalytic domain from Tylenchida.

In case of mixed phase intron 7, phase 1 was observed only for *Aphelenchus avenae*, a fungivorous nematode that can feed on root hair or epidermal cells of plants as well. Contrary to all other taxa investigated here, *A. avenae* does not belong to the order Tylenchida, though it is included in the infraorder Tylenchomorpha ( [33]). A phase 0 variant of intron 7 is found among a subset of the type A catalytic domains: the ones present in *Meloidogyne incognita* (with one exception: *Minc 19090a*), and in *Pratylenchus vulnus.* Among the 47 taxa harboring mixed phase intron 17, 13 were in phase 1, and 34 in phase 0. Phase 1 appeared to be a typical characteristic for type B and type C catalytic domains of cyst nematodes. Hence, it was also present in *eng3* and *eng4* from *G. rostochiensis*, two otherwise highly distinct cellulases. The only other case of an intron 17 in phase 1 was observed for *eng-1B* from the banana root nematode *Radopholus similis* (family Pratylenchidae)*.* This could be seen as a confirmation of a recent SSU rDNA-based analysis suggesting a (unexpected) close relatedness between *Radopholus* and cyst nematodes [20].

## Conclusions

Addition of 45 new genomic sequences from the catalytic domain of cellulases from plant parasitic members of the order Tylenchida, followed by phylogenetic analyses further develops our understanding of the evolution of cellulases within a nematode order that harbors most economically high impact plant parasites. Three distinct types of catalytic domains were distinguished, and we hypothesize that types of catalytic domains reflect distinct substrate preferences. Numerous plant parasitic nematode species were shown to harbor two types of GHF5 cellulases. *Heterodera glycines,* soybean cyst nematode, is the only example of a plant parasite equipped with all three types of catalytic domains distinguished so far.

All Clade 12 members of the phylum Nematoda analyzed to date harbor one or multiple GHF5 cellulases. This also holds for basal representatives that are not fully dependent on plants as sole food source. *Aphelenchus spp.*, on the basis of SSU rDNA data suggested to be sister to all Tylenchida [23], is primarily mycetophagous (fungal cell walls do not contain cellulose), but can also grow and multiply on various plant species [34]. It is noted that the necromenic nematode species *Pristionchus pacificus* (Clade 9, Diplogasteridae) is harbouring seven cellulase genes all belonging to GHF5 ( [35]). Hence, although GHF5 cellulases are widespread within Clade 12, they are not exclusively present in this clade.

The family Anguinidae harbours the most ancestral representatives of the order Tylenchida included in this paper*,* and *Ditylenchus destructor,* a member of this family and the causal agent of dry rot in potato tuber, is also known to feed on fungal hyphae. Recently, another early branching representative of the Tylenchida, *Deladenus siridicola -* a nematode with a mycetophagous and an insect parasitic life cycle, was shown to harbour GHF5 cellulases (dr. Bernard Slippers and co-workers, pers. comm.). Hence, all members of Clade 12 seem to harbor GHF5 cellulases, even ones that according to literature do not feed on plants. Presuming GHF5 cellulases were indeed acquired by lateral gene transfer, the most parsimonious explanation of the current cellulase tree would be the acquisition of such a gene by an ancient representative of the Pratylenchidae. Though we realize that current datasets are too fragmented to make a strong statement, our results are compatible with a scenario in which a GHF5 cellulase was acquired by the common ancestor of *Aphelenchus* and all Tylenchida, followed by one or multiple gene duplications and subsequent diversification.

## Competing interests

The authors have declared that there are no competing interests.

## Authors’ contributions

KR-M and JH designed this study, HvM collected and identified the biological material. KR-M, HRM, SvdE carried out the molecular work. KR-M and PM did the phylogenetic analysis. KR-M, GS, JB and JH contributed to data analyses and preparation of the manuscript. All authors read and approved the final manuscript.

## Supplementary Material

Additional file 1 **Table S1.** List of GHF5 endoglucanase sequences from plant parasitic nematodes from public databases used in this paper. Click here for file

Additional file 2 **Table S2.** Schematic overview of the (predicted) introns in genomic sequences from the GHF5 endoglucanase genes. For the intron identifiers, we adhered to the nomenclature proposed by Kyndt et al. (2008) [22]. Identifiers such as 1½ and 5½ were used for novel introns positioned between introns 1 and 2, 5 and 6, etc. The colour scheme for the phase of the introns is explained below this Table. Length of the introns (in bp) is given inside each box. Click here for file
