# Supplementary material for: Rather than by direct acquisition via lateral gene transfer, GHF5 cellulases were passed on from early Pratylenchidae to root-knot and cyst nematodes
Source: BMC Evol Biol. 2012 Nov 21;12:221. doi: 10.1186/1471-2148-12-221 (PMC3575306; doi:10.1186/1471-2148-12-221)
Supplement: Additional file 1 — Table S1. List of GHF5 endoglucanase sequences from plant parasitic nematodes from public databases used in this paper. [file 1471-2148-12-221-S1.pdf]

Supplementary Table 1. List of GHF5 endoglucanase sequences from plant parasitic nematodes from public databases used in this paper.

Taxon/cellulase name	GenBank Acc No	Data available	Fragment used for phylogenetics	Number of introns within CD1-CD6 region	Intron positions and lengths within CD1-CD6 region
**Nematoda**					
Aphelenchus avenae					
Aa-eng-1	AB495300	DNA, cDNA	CD1-CD6	5	SQ-WM 319; EAG 47; LQ-VD 40; TGE 43; KWF 85
Aa-eng-2	AB495302	DNA, cDNA	CD1-CD6	5	SQ-WM 337; EAG 44; LQ-VD 41; TGE 43; KWF 73
Ditylenchus africanus					
Da-eng-1	EU180235	DNA, cDNA	CD1-CD6	4	IQ-AL 67; FTQ 110; SQ-DV 103; NQ-IS 183
Ditylenchus destructor					
Dd-eng1-b	FJ430142	cDNA	CD1-CD6	-	-
Dd-eng-2	FJ374266	cDNA	CD1-CD6	-	-
Meloidogyne arenaria					
Ma-eng-1	AF323097	partial DNA	ENG1-ENG2	2	QA-IN 230;NK-GV 47;
Ma-eng-2	AF323098	partial DNA	ENG1-ENG2	2	QA-IN 355;NK-GV 46;
Meloidogyne chitwoodi					
Mc-eng-1	JN052069	cDNA	CD1-CD6	-	-
Mc-eng-2	JN052070	cDNA	CD1-CD6	-	-
Mc-eng-3	JN052071	cDNA	CD1-CD6	-	-
Meloidogyne hapla					
Mh-eng	AY277718	partial cDNA	ENG1-ENG2	-	-
Mh-eng-1	AF323096	partial DNA, cDNA	ENG1-ENG2	2	QA-IE 58; NN-GA 41;
Meloidogyne incognita					
Mi-eng-1a	AF323087	DNA, cDNA	CD1-CD6	3	KGS 369; QA-IE 71; NK-GA 45;
Mi-eng-2(a)	AF323088	DNA, cDNA	CD1-CD6	5	NV-VR 216; IA-NG 115; LP-VI 240; LMY 297; DFW 120;
Mi-eng-2(b)	AF323086	cDNA	CD1-CD6	-	
Mi-eng-3	AY422836	cDNA	CD1-CD6	-	
Mi-eng-4	AY422837	cDNA	CD1-CD6	-	
Mi9446a	Danchin et al.	cDNA	CD1-CD6	3	KGS; QA-IE; NK-GA;
Mi168	Danchin et al.	cDNA	CD1-CD6	3	KGN; KA-KE; DN-NV;
Mi3286	Danchin et al.	cDNA	CD1-CD6	3	KGN; KA-KE; DN-NV;
Mi14048	Danchin et al.	cDNA	CD1-CD6	3	KGN; KA-KE; DN-NV;
Mi169a	Danchin et al.	cDNA	CD1-CD6	3	KGN; KA-KE; NN-NV;
Mi3287	Danchin et al.	cDNA	CD1-CD6	3	KGN; KA-KE; NN-NV;
Mi14047a	Danchin et al.	cDNA	CD1-CD6	3	KGN; KA-KE; NN-NV;

Mi13221a	Danchin et al.	cDNA	CD1-CD6	3	KGS; QA-IE; NK-GA;
Mi9298a	Danchin et al.	cDNA	CD1-CD6	3	KGS; QA-IE; NK-GA;
Mi10405	Danchin et al.	cDNA	CD1-CD6	6	KLLR, NV-IR; ID-NG; LE-AI; TLY; KFL;
Mi18711	Danchin et al.	cDNA	CD1-CD6	5	NV-VR; IA-NG; LP-VI; LMY; DFW;
Mi19090a	Danchin et al.	cDNA	CD1-CD6	4	NI-IR; LW-AI; IMH; DFW;
Mi13614	Danchin et al.	cDNA	CD1-CD6	5	NV-IR; IE-NG; MK-AI; LMY; FLY;
Mi2089a	Danchin et al.	cDNA	CD1-CD6	5	NV-VR; MT-NG; LA-RV; LLY; DFW;
Meloidogyne javanica					
Mj-eng-1	AF323099	partial DNA	ENG1-ENG2	2	QA-IN 350; NK-GV 47;
Mj-eng-2	AF323100	partial DNA	ENG1-ENG2	2	QA-IN 457; NK-GV 46;
Mj-eng-3	AM231138	cDNA	CD1-CD6	-	-
Heterodera schachtii					
Hs-eng-1	AJ299386	cDNA	CD1-CD6	-	-
Hs-eng-2	AJ299387	cDNA	CD1-CD6	-	-
Heterodera avenae					
Ha-eng-1a	FJ839965	cDNA	CD1-CD6	-	-
Heterodera glycines					
Hg-eng-1	AF052733	DNA, cDNA	CD1-CD6	5	VK-AL 85; EA-VK 49; KK-VI 52; LMY 297; NGL 78;
Hg-eng-2	AF052734	DNA, cDNA	CD1-CD6	5	VK-AL 388; KA-IE 247; KK-VI 45; LMY 297; KGL 57;
Hg-eng-3	AF056048	DNA, cDNA	CD1-CD6	5	VK-AL 387; KA-IE 247; KK-VI 45; LMY 297; KGL 57;
Hg-eng-4	AY325809	DNA, cDNA	CD1-CD6	5	VK-AL 198; KA-IE 247; KK-VI 46; LMY 297; KGL 57;
Hg-eng-5	AY336935	DNA, cDNA	CD1-CD6	0	-
Hg-eng-6	AY163572	DNA, cDNA	CD1-CD6	7	KGT 920; DI-VRA333; EQ-EY 47; CIY 259; VK-VI 160; IMY 120;NWY 95
