# Supplementary material for: Rather than by direct acquisition via lateral gene transfer, GHF5 cellulases were passed on from early Pratylenchidae to root-knot and cyst nematodes
Source: BMC Evol Biol. 2012 Nov 21;12:221. doi: 10.1186/1471-2148-12-221 (PMC3575306; doi:10.1186/1471-2148-12-221)
Supplement: Additional file 2 — Table S2. Schematic overview of the (predicted) introns in genomic sequences from the GHF5 endoglucanase genes. For the intron identifiers, we adhered to the nomenclature proposed by Kyndt et al. (2008) [22]. Identifiers such as 1½ and 5½ were used for novel introns positioned between introns 1 and 2, 5 and 6, etc. The colour scheme for the phase of the introns is explained below this Table. Length of the introns (in bp) is given inside each box. [file 1471-2148-12-221-S2.pdf]

Supplementary Table 2. Schematic overview of the (predicted) introns in genomic sequences from the GHF5 endoglucanase genes. For the intron identifiers, we adhered to the nomenclature proposed by Kyndt et al. (2008) [20]. Identifiers such as 1½ and 5½ were used for novel introns positioned between introns 1 and 2, 5 and 6, etc. The colour scheme for the phase of the introns is explained below this Table. Length of the introns (in bp) is given inside each box.

			CD1											ENG1								CD4				ENG2		CD6		Linker	CBM2
Catalytic domain type	GenBank ID	Cellulase\Intron	1	1½	2	3	4	4½	5	5½	6	7	8	9	10	11	12	12½	13	14	15		15½	16	17	18	19	20	21	22	23
C	JN052066	Gp-eng-3													KA-IE 50				KK-VI 64*					LMY 101	NGL 48						
C	JN052067	Gp-eng-4			VK-AL 522										KA-LE 41				KK-VI 52*					LMY 145	NGL 48						
C	AY336935	Hg-eng-5																													
C	EF693943	Rs-eng-3			VK-QI 54																				LGL 113				NGV 50		
B	AF323098	Ma-eng-2													QA-IN 355										NK-GV 46*						
B	AF323097	Ma-eng-1													QA-IN 230										NK-GV 47*						
B	JN052024	Mard-eng-1	KDN 343												KS-IE 112										NK-GI 46*						
B	JN052025	Mart-eng-1	KGT 70												KA-IE 66										NK-GV 69						
B	JN052026	Mic-eng-1													QA-II 330																
B	JN052027	Mic-eng-2													QA-II 330																
B	AF323096	Mh-eng-1													QA-IE 58										NN-GA 41						
B	AF323087	Mi-eng-1a	KGS 369												QA-IEF 71										NK-GA 45				KNTGN 47	TGN 49	G-QQ 59
B	AF323099	Mj-eng-1													QA-IN 350										NK-GV 47*						
B	AF323100	Mj-eng-2													QA-IN 457										NK-GV 46*						
B	JN052028	Pcon-eng-1												SQ-AI 55											NK-GV 80						
B	JN052035	Pp-eng-3												SQ-AI 526											NK-GV 397						
B	JN052036	Pp-eng-4												SQ-AI 58											NK-GV 50						
B	JN052038	Pp-eng-6												SQ-AI 58											NK-GV 50						
B	JN052041	Ppr-eng-3												SQ-AI 58																	
B	EU176871	Pc-eng-1																							NK-KV 48*				NGV 46		56
B	JN052040	Ppr-eng-1																													
B	JN052043	Ppr-eng-4																													
B	JN052042	Ppr-eng-5																							NK-KV 55						
B	JN052044	Ppr-eng-6																							NK-KV 51						
B	JN052047	Pv-eng-1																							NT-KA 52						
B	JN052049	Pv-eng3																							NT-KA 52						
B	JN052050	Pv-eng-4																													
B	JN052053	Pv-eng-7																							NK-KV 53						
B	JN052054	Pv-eng-8																							NK-NV 303*						
B	JN052055	Pv-eng-9																							NK-KV 57						
B	JN052056	Pv-eng-10																							NK-KV 57						
B	JN052032	Pn-eng-1													QA-IA 56																
B	JN052033	Pn-eng-2													QA-IA 56																
B	JN052034	Pn-eng-3													QA-IA 56																
B	JN052031	Pcr-eng-1													QA-EA 53										NN-GL 46						
B	JN052030	Pcr-eng-2													QA-EA 52										NN-GL 44						
B	JN052029	Pcr-eng-3													QA-VT 50										NN-GI 44						
B	JN052037	Pp-eng-5	SGT 146												QA-VAF 109																
B	JN052045	Pt-eng-1													QA-IN 43										NN-GA 48						
B	JN052046	Pt-eng-2													QA-IN 43										NN-GA 48						
B	JN052051	Pv-eng-5													QA-VA 409										DN-GL 100						
B	JN052052	Pv-eng-6													?-VA										DN-?						
B	JN052061	Hgr-eng-1													SA-AS 137										NN-GI 85						
B	JN052062	Hgr-eng-2													QA-VS 60										NA-GL 81						
B	JN052063	Hgr-eng-3													SA-VS 137										NN-GI 85						
B	JN052057	Hl-eng-1						LA-GY 27																							
B	JN052058	Hl-eng-2													SA-VS 73																
B	JN052059	Hl-eng-3													QA-VE 459										NN-GL 63						
B	JN052060	Hl-eng-4													SA-VT 53										TK-GL 70						
B	JN052064	Gp-eng-1			VK-AL 563										KA-AE 47				KK-VI 53*												
B	JN052065	Gp-eng-2													KA-IE 50				KK-VI 64*					LMY 101	NGL 48						
B	AF056110	Gr-eng-1			VK-AL 308										KA-IE 53				KK-VI 63*					LMY 101	NGL 93			AN-VG 51	TGV 53		AG-TT 214
B	AF056111	Gr-eng-2			VK-AL 742										KA-VE 49				KK-VI 56*					LMY 102	NGL 67			AN-VG 51	TGV 49		
B	AF408154	Gr-eng-3			VN-RI 354				TQ-RS 64						SA-KE 62				KT-MV 111						LGL 50				NGV 113		
B	AF408157	Gr-eng-4			VN-RI 271				TQ-KS 65						SA-KE 61				KT-MV 111						LGL 50				NGV 114		
B	AF052733	Hg-eng-1			VK-AL 85										EA-VK 49				KK-VI 52*					LMY 297	NGL 78*			AN-VG 50	TGV 46		SG-TT 76
B	AF052734	Hg-eng-2			VK-AL 388										KA-IE 247				KK-VI 45					LMY 297	KGL 57			AN-VG 133	TGV 150		
B	AF056048	Hg-eng-3			VK-AL 387										KA-IE 247				KK-VI 45					LMY 297	KGL 57			AN-VG 133	TGV 150		
B	AY325809	Hg-eng-4			VK-AL 198										KA-IE 247				KK-VI 46					LMY 297	KGL 57			AN-VG 133			
B	EF693940	Rs-eng-1A																													SG-TT 48
B	EF693941	Rs-eng-1B				QQ-LK 106											TK-VS 46								NGLP 72			ST-VG 44	NGV 106		SG-TT 247
B	EF693942	Rs-eng-2																											NGV 56		
A	AF323088	Mi-eng-2a					NV-VRA 216					IA-NG 115								LP-VI 240				LMY 297		DFW 120			NGI 39		
A	JN052048	Pv-eng-2					NV-IR 55			ES-AP 25		IA-NG 90*								IK-AI 56					IN-GL 29*	LWW 132					
A	JN052039	Ppr-eng-2					DI-VR 274						CMY 51							IK-AI 84				IMY 46		EWY 76					
A	AY163572	Hg-eng-6	KGT 920				DI-VR 333				EQ-EY 47		CIY 259							VK-VI 160				IMY 120		NWY 95					
Not yet classified	AB495300	Aa-eng-1		SQ-WM 319								EAG 47						LQ-VD 40					TGE 43			KWFA 85					
Not yet classified	AB495302	Aa-eng-2		SQ-WM 337								EAG 44						LQ-VD 41					TGE 43			KWFA 73					
Not yet classified	EU180235	Da-eng-1			IQ-AL 67											FTQ 110					SQ-	DV 103					NQ-IS 183				SG-QT 149

(light green)	=	no introns could be found in this protein fragment
(light orange)	=	phase 0
(yellow-orange)	=	phase 1
(orange)	=	phase 2
*	=	GC-AG type
Underlined	=	sequences generated in this study
?	=	unknown data
